# What is the problem of dependency? Dependency work reconsidered

**DOI:** 10.1111/nup.12327

**Published:** 2020-09-15

**Authors:** Simon van der Weele, Femmianne Bredewold, Carlo Leget, Evelien Tonkens

**Affiliations:** ^1^ University of Humanistic Studies Kromme Nieuwegracht 29 Utrecht 3512 HD The Netherlands

## Abstract

Dependency is fundamental to caring relationships. However, given that dependency implies asymmetry, it also brings moral problems for nursing. In nursing theory and theories of care, dependency tends to be framed as a problem of self‐determination—a tendency that is mirrored in contemporary policy and practice. This paper argues that this problem frame is too narrow. The aim of the paper is to articulate additional theoretical ‘problem frames’ for dependency and to increase our understanding of how dependency can be navigated in practices of long‐term care. It does so by way of an empirical ethical analysis of how care professionals tackle the problem of dependency in group homes for people with intellectual disabilities. The paper refers to these practices of mitigating the problem of dependency as ‘dependency work’, a phrase borrowed from Eva Kittay. The analysis of dependency work suggests that for care professionals, dependency is a threefold problem: one of self‐determination, one of parity and one of self‐worth. These findings suggest that patient autonomy cannot be a full solution to the problem of dependency in long‐term care relations. But they also show that dependency as such is not a problem that can be *solved*, as attempts to mitigate it only serve to tighten the dependency relationship further. This is the paradox of dependency work.

## 1 INTRODUCTION

Dependency is a fundamental concept for the theory and practice of nursing and caring (Collins, 2015; Delmar, 2013; Engster, 2019; van der Weele, accepted for publication). By definition, caring relationships are relationships of dependency, too. This has been recognized by foundational thinkers of nursing studies and care ethics alike. Virginia Henderson, for instance, defined the nurse's function as ‘to assist the individual, sick or well, in the performance of those activities contributing to health or its recovery (or to peaceful death) that he would perform unaided if he had the necessary strength, will or knowledge’ (Henderson, 1964, p. 64). Henderson thus assumed nursing to spring forth from a relationship of dependency between the caretaker and the care recipient (Dorothea Orem (1959) spoke of a ‘self‐care deficit’). Similarly, Joan Tronto writes that ‘care arises out of the fact that not all humans or others or objects in the world are equally able, at all times, to take care of themselves’ (1993, p. 145). A caregiver meets a need that the care recipient cannot meet herself. It is in this sense that care implies dependency, and this dependency, as many authors have noted, is fundamental to the human condition itself, as all of us are dependent on others to survive and flourish, at least in some stages of life (Butler, 2010; Engster, 2019; Fineman, 2017; Kittay, 1999; MacIntyre, 1999).^1^ Dependency is not the only way to characterize the care relationship—for example, caregiver and care recipient can in many ways be said to be *inter*dependent. However, as Eva Kittay observes, ‘interdependence begins with dependence’ (1999, p. xii): a more fundamental dependency precedes any possibility of interdependence, and this dependency is our focus here.

If dependency is foundational to nursing and caring, it also makes the caring relationship morally charged. Given that care implies dependency, it implies asymmetry too (Collins, 2015). This is what Tronto calls ‘the fact of inequality in relations of care’ (1993, p. 145). If a caregiver meets a need that the care recipient cannot meet herself, their relationship is lopsided (Strandberg, Norberg, & Jansson, 2000). This lopsidedness turns dependency into a matter of power as well as of care and here arguably begin the ethics of caring. This is what we take Stacy Clifford Simplican to mean when she writes that ‘[o]ur dependence is both the foundation of and a problem for caring relationships’ (2017, p. 401). But what sort of problem? And for whom?

In this paper, we take a closer look at how dependency surfaces as a problem in long‐term care relationships. It is our impression that by care theorists and policy makers alike, dependency is predominantly framed as a problem of self‐determination. This is a helpful frame, as it illuminates how the power imbalance in care relationships can work to limit freedom and autonomy (Risjord, 2014). However, we also believe this frame is too limited, as there are other, distinct ways in which dependency can trouble care relationships. The aim of this paper is to articulate additional theoretical ‘problem frames’ for dependency to increase our understanding of how dependency can be navigated in practices of long‐term residential care.

We do so by exploring how dependency surfaces as a problem for care professionals. Empirical studies indicate that nurses think of their patient's dependency as a demanding responsibility (Piredda et al., 2020; Strandberg & Jansson, 2003). Our aim here is to investigate how care professionals navigate this responsibility in practice. We think that care theorists can learn a lot from care professionals by looking at how they work. Here, we follow Tronto, who claims that ‘care practices are critical. Practitioners in care practices attempt to improve the way that they are engaging in their practice, and such reflection makes them reflective about the practice’ (van Nistelrooij, Schaafsma, & Tronto, 2014, p. 489). Following Tronto, we suggest that the practices in which care professionals engage might have much to reveal about the problem dependency poses for caregiving and how to deal with it. Studying how care professionals navigate dependency, we contend, orients us towards a more differentiated account of the ‘problem’ of dependency, in which dependency arises not only as a problem of self‐determination, but also as a problem of parity and as one of self‐worth. However, since the frame of self‐determination is dominant both in care theory and policy, care professionals work on these problems tacitly, without an appropriate vocabulary. Teasing out these different problem frames, then, serves two ends: first, to further elaborate on the meaning of dependency as a keyword in care theory; and second, to provide a vocabulary for care professionals that accounts for the rich repertoire of strategies with which they tackle problems of dependency in practice.

A first suggestion for this vocabulary is to refer to such practices of tackling the problem of dependency as ‘dependency work’, a phrase we borrow from Kittay (1999). Kittay defines dependency work as ‘the task of attending to dependents’ (1999, p. 30). It covers the care for dependents in a broad sense, from washing and toileting to administration and insurance. Our argument, however, is that much of this work is not only geared towards relieving dependents of their needs; in addition, dependency work consists in navigating and mitigating the moral tensions of the dependency relationship themselves. Dependency work, then, also denotes the task of working with and against dependency. In what follows, we trace such practices of dependency work to arrive at a more diversified theory of the problem of dependency itself, by drawing on ethnographic research undertaken in group homes for people with intellectual disabilities (ID) in the Netherlands. But first, some words on methodology.

## 2 TRACING ‘GOODS’ IN CARE PRACTICES

In our investigation, we follow the ‘empirical ethics of care’ approach advanced by Pols (2013, 2015, 2019) and Ceci, Pols, and Purkis (2017). The central premise of this approach is that ‘[c]are practices have a normative orientation towards some kind of good’ (Ceci et al., 2017, p. 57). By caring for others, care professionals give expression to values, tastes and ideals: their work consists of ‘attempts to put something good into practice’ (2015, p. 83). The task an empirical ethics of care sets for itself is to study what these goods might be; how they might conflict; and how they pan out for the participants involved. This requires empirical attentiveness to care practices and to what care professionals say about these practices, as normativity expresses itself as much in practices themselves as in the vocalized intentions that underpin them. This is because practices are directed towards doing something perceived as good—regardless of whether it is actually achieved. Moreover, the goods expressed in care practices also hint at the problems these practices are meant to address. According to Pols, an ‘empirical ethics of care’ approach allows us to ‘attend to what kind of problems people encounter, how they may be solved, and which values are hence brought into being’ (2019, p. 59). In other words, reconstructing the goods care professionals pursue also brings into view the problems people attempt to solve by means of these goods. In what follows, we trace those goods that seem to be an answer to the ‘problem’ of dependency, because they somehow grapple with the asymmetries that form both ‘the foundation of and a problem for caring relationships’ (Simplican, 2017, p. 401). By articulating these goods, we also get an idea of what *kind* of problems dependency poses for practice. Note that our aim is not to establish whether or not care professionals *succeed* in shaping these goods or to evaluate their outcomes. Rather, we wish to spell out the moral logic by which care professionals seem to work and show how different logics provide different solutions for different problems of dependency.

Our site for examining the goods of care practices is long‐term care for people with ID. This type of care is diverse, covering aiding persons in activities of daily living as well as in navigating work, leisure and all kinds of relationships. Such care is often referred to as ‘assistance’ or ‘support’. This is no coincidence: calling such activities ‘care’ is an unpopular choice, as ‘care’ already evokes much of what makes ‘dependency’ suspicious to some (Kittay, 2011; Kröger, 2009; Morris, 1997). Indeed, much social scientific work on ID support treats dependency with distrust. A normative focus on promoting *independence* seems to render dependency as naturally antithetical to the goals of assistance (Hawkins, Redley, & Holland, 2011).^2^ When the word ‘dependency’ is invoked, it carries negative connotations. This makes ID care a fitting terrain of study, as dependency is contested from the outset, demanding a practical response from support workers (Meininger, 2001; Pols, Althoff, & Bransen, 2017; Wilson, Clegg, & Hardy, 2008). How care professionals (or ‘support workers’) traverse this field becomes a matter of empirical as well as theoretical interest.

The empirical material discussed below was collected during an ethnographic study carried out by the first and second author (respectively, a philosopher and an anthropologist) in a study into experiences of dependency in disability care in the Netherlands.^3^ The study was commissioned by the Dutch Ministry of Health and aimed to determine how ‘negative experiences of dependency’ can be limited or prevented. The bulk of our observations were made while shadowing care professionals in assisted living group homes for people with mild, moderate or severe ID. As we watched them (and occasionally helped them), we also asked questions about what they were doing and why they did so, resulting in thick descriptions of events and what they meant to those involved (McDonald & Simpson, 2014). We visited the group homes as relative outsiders, under conditions of what Elizabeth Quinlan calls ‘conspicuous invisibility’: a sense of being ‘there but not there’, present but not quite as active participants in care (Quinlan, 2008, p. 1480). The role of curious bystander permitted us to attempt an articulation of the ‘goods’ that tend to go unspoken in practice, by observing and conversing with the care professionals who enact them (Pols, 2008). While we strove to chronicle practices in a broad sense, including more structural practices on the level of protocol, bureaucracy and architecture, our focus was on daily interactions between caregivers and care recipients as the locus of everyday caring.

In what follows, we first describe how dependency has predominantly been framed as a problem of self‐determination by theorists of care. Then, we turn to practice, by enumerating three forms the problem of dependency seems to take in the care for people with ID. Each of these problems is implicit in the goods care professionals appear to pursue in their daily care practices. We group these goods in clusters that form three practical approaches to navigating the dependency relationship: one centred on ‘agentive’ goods, one on ‘equalizing’ goods, and one on ‘affirmative’ goods. We illustrate each with examples. Taken together, they form a repertoire for the practice of what we call dependency work: the practice of working with and against the problem of dependency in care relations.

## 3 DEPENDENCY AS A PROBLEM OF SELF‐DETERMINATION—IN THEORY

Given that dependency (and the power relationship it implies) suffuses the practice of caring with moral tensions, it is not surprising that many care theorists have considered dependency as a problem for care relations. In such work, dependency tends to get framed as a problem that has to do with (patient) self‐determination, (patient) freedom or (patient) autonomy. This argument comes in different guises, using different words. One such word is *paternalism* (Delmar, 2012; Kittay, 2019; Risjord, 2014; Tronto, 1993). Tronto speaks of paternalism when caregivers ‘come to accept their own account of what is necessary to meet the caring need as definitive’, resulting in diminished autonomy for the care recipient (1993, p. 145). Mark Risjord's bioethically informed understanding of paternalism is ‘to act for the patient's benefit but without the patient's consent’, again resulting in diminished autonomy (2014, p. 35). A second such word is *domination*. Kittay defines domination as the abuse of power inequalities that are endemic to dependency relations and which result in diminished freedom or autonomy (1999, pp. 33–36). For Kittay, the problem of domination extends to both parties in the dependency relation, as both the caregiver and the care recipient are vulnerable to face abuse by the other. Moreover, Kittay traces another form of domination in what she calls ‘secondary dependency’, which refers to forms of political, social and economic dependency faced by caregivers who bear the responsibility of (often unpaid or underpaid) care work (1999, p. 46). A third such word is *subordination*. Disability philosopher Anita Silvers conceives of dependency as created by paternalistic practices of care that consign persons with (physical) disabilities to ‘subordinated relationships’ of caring (1995, p. 43). For Silvers, the subordination of ‘patients’ (in her case, people with disabilities) is a product of care practices, rather than a given. Dependency, in other words, impedes freedom.

Paternalism, domination, subordination—what ties these words together is a concern about self‐determination, which is deemed to be at risk in relations of dependency. It was for this reason, perhaps, that nursing theorists like Virginia Henderson believed the main task of nurses was to ‘help [the patient] gain independence as rapidly as possible’ (1964, p. 63). Indeed, if dependency tends to get framed a problem of self‐determination, ‘independence’ is often invoked to signify a state of being in control. For instance, disability writer Jenny Morris (1997, p. 56) famously claimed that ‘[i]ndependence is not about doing everything for yourself, but about having control over how help is provided’, thus suggesting that the problem of dependency is a lack of control. Of course, since then, many theorists (including Morris herself) have challenged the inverse relationship between dependency and autonomy as Henderson seems to suggest it (Anderson & Honneth, 2004; Mackenzie & Stoljar, 2000; Meininger, 2001; Morris, 2001; Risjord, 2014). What matters here is that few theorists would deny that some forms of dependency relationships could pose a problem for securing (patient) autonomy.

This dominance of the self‐determination problem frame is mirrored in care policies in many countries in the Global North. European welfare states, for instance, have increasingly designed care policies around words such as independence and control (Newman & Tonkens, 2011). These concepts also posit a view of what sort of problem dependency poses: a problem of diminished self‐sufficiency and inordinate reliance on the state (Fraser & Gordon, 1994). If dependency entails diminished self‐sufficiency, it may hamper self‐determination. In such a political climate, dependency becomes a suspect condition, rife with negative associations (Schram, 2000). In other words, dependency is now a policy problem to be solved—and since it is perceived to be a problem of self‐determination, the solution is looked for in concepts such as patient autonomy, patient choice and patient control and independence (Mol, 2008) (tellingly, the empirical study we rely on for our arguments, on ‘negative experiences of dependency’, was originally commissioned by the Dutch Ministry of Health). To be sure, care theorists generally would not endorse the negative connotations dependency carries in contemporary care policy. To the contrary, their work often seeks to revalorize dependency by depicting it as integral to the human condition (Kittay, 1999). Nonetheless, what connects theory to policy here is the problem frame: dependency predominantly gets represented as a problem of self‐determination.

## 4 DEPENDENCY AS A PROBLEM OF SELF‐DETERMINATION—IN PRACTICE

Care professionals do not work in a state of pure autonomy. Their practices are influenced by the policy and organizational contexts in which they are embedded. In the Netherlands, true to the trend described above, this has meant a striving towards self‐determination and self‐reliance (Fenger & Broekema, 2019; Reinders, 2002). If care theorists and managers alike frame dependency as a problem of self‐determination, what does that mean for care in practice?

Given that the dominant problem frame amongst policy makers and managers is the frame of self‐determination, it should come as no surprise that care professionals, too, appear to engage with dependency as such. Whenever the topic of dependency came up in conversation, care professionals spoke of it in terms of a contrast with choice and control. One even described her work as looking for a ‘golden mean between control and dependency’. Dependency was something care professionals wanted to avoid or at least limit, in favour of self‐determination, which they often referred to as ‘independence’. This also shows in their practice. Care professionals constantly attempt to provide choices, both big and small. They eagerly encourage residents to carry out tasks themselves. They also look for inventive ways of letting residents have their say. We refer to such attempts as ‘agentive’ goods. The pursuit of agentive goods frames dependency as a problem of diminished self‐determination. Care professionals try to transfer some of their own ‘executive powers’ to the care recipient in order to provide opportunities to exert agency. *Choice*, *doing things yourself* and *giving a voice* are agentive goods. ‘We want them to live somewhat independently. That they can make their own choices’, one professional said. And another: ‘I think we give people freedom, let them choose. I always ask, can I come in? It's their home, after all.’

Care professionals keenly recognize that not everyone with ID can exert agency in the same way. How they exercise agentive goods depends on who they are assisting. For people with mild ID, care professionals tend to pursue agentive goods in as many life domains as possible, encompassing everyday decisions and actions as well as major life choices. For people with severe ID, the reach of agentive goods is more limited, focusing on concrete everyday activities such as eating and playing.

Care professionals get creative in finding ways to pursue agentive goods, even for people who have limited capacity for self‐determination. Take the example of eating. For people who eat independently, care professionals might structure choice.

Care professional Jessica^4^ asks Ireen which soup she wants to eat tonight: mushroom or tomato? Ireen opts for mushroom. Jessica helps Ireen put the soup in her microwave. After we leave Ireen's apartment, Jessica explains that she always gives Ireen two options, even if there are more soups to choose from. If she doesn't, choosing gets too complicated for Ireen, which can cause her to get anxious.

For people who do not eat independently, care professionals experiment in the vast space between eating independently and being fed. They might prepare a bite on a fork, hand the fork to a resident and let the resident pick up the fork to savour the bite; this works for people who cannot fork up a bite, but can raise their own fork to their mouths. Or they might prepare a bite on a fork, place the bite on a separate plate and let the resident fork up the bite from the plate with their own fork; this works for people who cannot prepare their own bite, but can fork up a bite and can raise their own fork to their mouths. In this way, care professionals fine‐tune the process of eating, splicing it into a number of tiny processes, each of which can be delegated to the resident in order to establish a sense of agency.

This last example also points towards a limitation of agentive goods. The ‘can’ of eating by yourself is not merely a matter of capacity, but also one of safety and risk. Residents who do not swallow well or who eat too quickly and who are therefore vulnerable to chocking cannot be left forking up their own meal without supervision. As Pols (2015) point out, sometimes goods are incompatible. In the present case, agentive goods such as choice and doing things yourself can be in conflict with other ones deemed equally important to the practice of caring, such as safety, health, cleanliness and well‐being (Askheim, 2003). In most cases, care professionals seek to find space for agentive goods within the boundaries set by these other goods. This might mean to supervise the pace of their eating or limit their smoking allowance.

Care professional Erik and his colleagues ration Huub's cigarette intake. Huub has a special box with various compartments. Each compartment contains two cigarettes. Every two hours, Huub drops by the office to pick up two cigarettes. ‘He wouldn't say: I don't want to smoke 30 cigarettes a day,’ Erik explains. ‘But neither would he enjoy coughing and wheezing all day.’

Things get more complex in life domains like love and work. In these instances, pursuing agentive goods triggers questions about responsibility and influence, which bear heavy on care professionals.

Since some weeks, Ada has a boyfriend. She has asked care professional Pauli whether he can stay over next Saturday. Pauli tells me she is unsure. She doubts the situation will feel safe enough for Ada to enjoy. ‘Who am I to decide whether or not your relationship is too short for that?’, she wonders. She wants to give residents space to make mistakes. But when is such space in order? And when do residents need to be shielded from their mistakes? I ask Pauli what she intends to do. She says if Ada does not decide by herself that it's too soon in her relationship, Pauli will advise Ada to wait a while longer.

This example shows how the logic of agentive goods is salient even in cases when care professionals have a clear agenda of their own: Pauli hopes Ada will figure out the right thing to do *by herself*, but if she does not, Pauli will attempt to steer her towards the course of action she prefers.

If the problem of dependency is one of self‐determination, care professionals attempt to mitigate this problem by pursuing agentive goods. But agentive goods cannot diminish the dependency relationship itself. Rather, care professionals see it as their role to aid residents in their exercise of agency.

Care professional Manuela tells me about a resident who had been wanting to quit her job for a while. Manuela had repeatedly advised against this, as she thought a stable job was best for her. Then, Manuela and the resident had a conversation about making choices. Manuela explained what it meant to make a choice. The resident mentioned her work: quitting was *her* choice. ‘But you don't approve, right?’ At this point, Manuela gave in. She did not agree, but she believes care professionals should teach residents that they can choose what they want. The more you use terms like choice and decision, the more residents remember them, think about them, and understand them.

Understood in this way, self‐determination is a constant learning project. The pursuit of agentive goods facilitates this process, which makes the care professional all the more indispensable for enabling self‐determination in the first place.

## 5 ALTERNATIVE PROBLEM FRAMES FOR DEPENDENCY

Throughout our fieldwork, care professionals were mostly tackling the problem of dependency by way of agentive goods. In this sense, the concern of care professionals appears to mirror the concern of care theorists, who tend to frame dependency as a problem of self‐determination, too. Given the embrace of ideals like self‐determination and self‐reliance amongst policy makers and care managers alike, this is unsurprising (Mol, 2008). The institutional context does not exhaust the possibilities of action, though, as care professionals can (and will) do things no one might expect (or even want) them to do. From these practices can also emerge different takes on what the ‘problem’ of dependency might be. In what follows, we discuss two other clusters of goods, which frame the problem of dependency differently. Distinguishing these goods from ‘agentive’ ones will show that the problem frame of self‐determination is too limited.

### 5.1 Equalizing goods: dependency as a problem of parity

The first cluster of these goods we call ‘equalizing’. The pursuit of equalizing goods marks dependency as a problem of diminished parity in the care relationship. This expresses itself in trying to balance the dependency relationship by allowing for more equal and diverse interactions to take place between caregiver and care recipient. We deem goods such as *participation*, *reciprocity* and *affection* equalizing. We group these goods together because all are geared towards forging a more informal and (sometimes) more mutually enriching caring relationship, bridging ‘professional distance’ towards what might be called ‘professional proximity’.

Cultivating a relationship of professional proximity happens through subtle gestures of mutuality, hinting at a shared life. Equalizing goods can be found in moments of informal contact, like smoking a cigarette together, watching a soccer match together or sharing a meal. Such moments break down the hierarchical relationship dependency is perceived to set in place.

Care professional Julius has his meal with the residents. He tells me some colleagues bring their meal from home, but he doesn't; he thinks that's improper. I ask him why. ‘I am no more than they are,’ he answers.

In this professional's logic, participation serves to equalize. The same holds true for reciprocity. Some care professionals attempt to facilitate moments of reciprocity, both between professionals and care recipients and between care recipients and other people.

Care professional Wieteke is rubbing hand cream onto Francien's hands. Care professional Rian walks by. ‘Nice!’, she exclaims. She tells Francien she could return the favour to Wieteke. Francien begins to smile. She starts rubbing Wieteke's hands.

By promoting such moments of reciprocity, care professionals give residents a chance to reverse the care relationship and increase their sense of parity.

The pursuit of equalizing goods is perhaps most visible in the banter that we found to typify many interactions in everyday ID care. By engaging into humorous exchanges, care professionals can participate in the experiential world of residents and create reciprocal connections, even if briefly. Jokes soothe and defuse tensions between caregivers and care recipients. They release some of the pressure that the inequality inherent to dependency relationships brings to everyday interactions, as they allow carers to ridicule their authority and care recipients to rebel against their carers.

Over dinner, residents are speaking about summer holidays. Bram jokingly encourages the assistants to go on holiday too: for twelve weeks, if they wish. Gijs agrees: he wouldn't mind them going on holiday for fourteen weeks. Laughing, Jos says the assistants can go on holiday forever. Valerie says she wouldn't mind going on holiday, but asks who's going to pay. It's like going on retirement – it's not free!

Humour, then, is one strategy to pursue equalizing goods. As mentioned, these goods often centre on achieving parity through informal gestures of mutuality. But even if these interactions are informal, they are not necessarily spontaneous: care professionals might pursue equalizing goods deliberately in order to affect their residents’ perception of them.

Erik is originally from Germany. When the German national soccer team is playing, residents sometimes let him watch the matches in their room. ‘The clients like it,’ he says. ‘They see you differently then.’ He explains: ‘I try not only coming to check in on them, making sure they follow the rules. I try to come just for a chat. So that the clients don't get startled whenever I’m around.’

Through casual interactions, Erik attempts to show he can be ‘one of them’ and foster a sense of trust amongst the residents for whom he cares. To be sure, this is one way of striving for parity. But it also hints at what is professional about ‘professional proximity’. Trust is instrumental in doing a better job at assisting, as residents will be quicker to confide and share. Working on equalizing goods, then, can be an end in itself, but also a means towards another goal. In the words of one assistant: ‘As assistant, you want clients to trust you… you want them to keep on talking to you about their problems.’ In this sense, even if equalizing goods aim to tackle the problem of dependency by cultivating parity, this is not to do away with the dependency relationship, but to have it function as well as possible.

Equalizing goods attempt to mitigate the problem of dependency by establishing a sense of mutuality in the care relationship. There are limits to implementing these goods, however, and these are set by contrasting goods also pursued by care professionals, such as independence and (obviously) professional distance. As one care professional comments, ‘you do fuse together in a way.’ Not all care professionals consider this desirable—nor do all people with ID. How professionals deal with the tensions between these goods is often a matter of what they take their own job description to be. This might be one reason why equalizing goods are not as dominant as agentive ones.

### 5.2 Affirmative goods: dependency as a problem of self‐worth

The third cluster of goods we wish to distinguish consists of ‘affirmative goods’. These goods frame dependency as a problem of diminished self‐worth. This expresses itself in trying to foster sustain a positive self‐image in the care recipient. Some affirmative goods we found are *praise* and *confidence*. By pursuing such goods, care professionals appear to promote feelings of confidence and competence amongst residents, thus counteracting the sense of inadequacy dependency is perceived to engender.

Of all three clusters of goods, the pursuit of affirmative goods is the most casual and habitual we observed. Care professionals ceaselessly embellish their interactions with residents with compliments. Actions well‐executed, feelings well‐expressed and ideas well‐conceived can usually count on generous responses from care professionals, who seem eager to assert their delight in witnessing their residents’ achievements. So, care professionals might praise a piece of art, a successful card payment or a well‐administered Band‐Aid. Or they might compliment a resident for showing initiative to clean, for being honest to a fellow resident, or for making a good suggestion during a residents’ meeting. In everything, a client does or says surfaces an opportunity for recognition.

Two points are of note here. First, in some cases, whether or not the praise is sincere appears to be of secondary importance.

Stefan folds his hand to resemble a microphone and begins to sing. The words are difficult to make out, but the melody resembles a Dutch folk song. His booming voice fills the space. The other resident in the living room gets up and leaves. Care professional Wendy attempts to interrupt him. ‘Very pretty, Stefan,’ she calls out, and a little while later, ‘beautiful!’ She also begins applauding Stefan before the song is over. Stefan stops only after finishing his song. Wendy applauds once more and compliments his performance.

In this example, the care professional's praise might also serve an attempt to cut Stefan short (quiescence is what Wendy is after). What matters is that she does so by complimenting, rather than admonishing.

Second, in some cases, whether or not the praise is *understood* as such appears to be of secondary importance, too. Care professionals are just as likely to compliment people with severe ID who are nonverbal.

Care professional Jantine explains Linda can eat anything. ‘She eats everything, I am not sure she is able to experience flavour. Some residents can't.’ Then she turns to Linda. ‘Delicious, right, girl? What you don't like to eat still has to be invented.’

Jantine turns what might be perceived as a shortcoming into a virtue, for which Linda may feel a sense of pride; whether or not Linda is aware of this virtue herself, is not what matters to Jantine.

Giving compliments forms part of a larger project of boosting residents’ confidence. Another manifestation of this project can be found in affirmative ‘pep‐talks’ care professionals are prone to give residents. Providing words of encouragements, these affirming mini‐speeches seek to empower residents.

Erik and Daisy are talking about show dancing and swimming, two activities she is considering taking up. She asks Erik whether she has to do both. ‘You are your own boss. You are the boss over what you do at night. You are the boss over whether you feel like swimming and you are the boss over whether you feel like show dancing’, Erik replies seriously. Daisy nods.

Such mini‐speeches remind residents of their agency and strength. While they are mantras of self‐determination, their purpose seems to be to grant residents confidence to act on their capacity for self‐determination. Nonetheless, it is clear from these examples how the pursuit of agentive goods and affirmative goods could imbricate. For these care professionals, cultivating confidence might be a method for helping residents achieve self‐determination.

The pursuit of affirmative goods suggests that dependency ought to be seen as a problem of diminished self‐worth. It attempts to mitigate this problem by attempting to give residents a sense of confidence. As with agentive and equalizing goods, implementing affirmative goods does not undo the dependency relationship as such; rather, it affirms it, as care professionals become a main source of confidence for the residents for whom they care.

## 6 CONCLUSIONS: DEPENDENCY WORK RECONSIDERED

The clusters of agentive, equalizing and affirmative goods we outline above provide us with three distinct frames on the problem dependency can be said to engender in long‐term care: diminished self‐determination, diminished parity and diminished self‐worth. In addition, these clusters of goods also tentatively form three approaches for dealing with these different problems of dependency: diminished self‐determination can be prevented or mitigated by pursuing agentive goods; diminished parity by pursuing equalizing goods; and diminished self‐worth by pursuing affirmative goods. While we have gleaned these problem frames from practices in long‐term residential care for people with ID, we think they apply more broadly to long‐term (residential) care settings in general or to any care setting in which caregiver and care recipient enter a prolonged relationship of care. We wish to draw several conclusions from these empirical findings.

The first of these centres on the notion of dependency work. Even if their work involves more than the pursuit of agentive, equalizing and affirmative goods, it is striking just how much (and how creatively) care professionals busy themselves with the problem of dependency. Taken together, these practices of mitigating problems rooted in the dependency relationship allow for a specification of what Kittay (1999, p. 30) calls dependency work: ‘the task of attending to dependents.’ We think that much of what Kittay would call dependency work is geared towards more than simply relieving dependents of their needs; many of the practices we witnessed were in fact about navigating the moral tensions of the dependency relationship itself. That is to say: in our rendition, dependency work consists of those practices that seek to mitigate the problems that might arise from the dependency relationship; or, put differently, that seek to render dependency in long‐term care more bearable. Even if they are unaware of it, this is a task in which most care professionals appear engaged.

However, while care professionals may attempt to mitigate problems of dependency, dependency work will not *solve* the dependency relationship itself. Quite the opposite: agentive, equalizing and affirmative goods each tighten the dependency relationship further. This we might call a paradox of dependency work: tackling the problem of dependency actually brings an intensification of the dependency relationship. The purpose of dependency work is not to undo dependency, but to make that relationship function as well as possible. This conclusion gives credence to arguments put forth by Reinders (2010) and Piredda et al. (2015), who argue that the quality of the care relationship determines the quality of care and the experience of dependency.

A caveat is in order here. Our aim has been to spell out the moral logic by which care professionals seem to work—not to evaluate the quality of their care practices as such. While these three clusters of good certainly appear to exist in practice, we have not said how often they are used or fail to get used while they could or should have been. We also do not reflect on whether these goods ought to be implemented differently or more radically. Care professionals are usually well aware of their shortcomings; it is hardly up to us to tell them they ought to do better. Nonetheless, the analysis presented here might serve to make care workers and nurses alike aware of the repertoire they draw from when they go about their dependency work; a repertoire richer and more complex than the pursuit of autonomy or independence.

This brings us to the second conclusion, on the ‘problem’ of dependency. As we have shown, the problem of dependency has predominantly been framed in terms of diminished self‐determination, in theory and practice alike. Care theorists have done so by drawing on a vocabulary of paternalism, domination and subordination. Care professionals do so by pursuing agentive goods. However, while care professionals widely profess (and are certainly most eloquent about) their intentions when it comes to stimulating self‐determination, their caring repertoire is in fact richer: their care practices tacitly seem to pursue different goods as well, which deal with different problems, i.e. diminished parity and self‐worth. Our findings thus suggest that the problem of dependency has generally been too narrowly construed as a problem of self‐determination, downplaying the effects of dependency on equal social interactions and the care recipients’ self‐worth. The problem of dependency is in fact (at least) a threefold problem, with (at least) three approaches to handling it—agentive, equalizing and affirmative goods. The care professionals we followed were equipped with the means for handling this more diverse set of issues but lacked a vocabulary for expressing this. Our analysis of these practices in terms of the pursuit of goods is an attempt to articulate a vocabulary.

As Pols (2015, p. 82) notes, goods can be in conflict. If the problem of dependency turns out to be multifaceted, the approach for dealing with one might not work for another. ‘Patient autonomy,’ then, is probably not a complete solution to the problems dependency might engender in long‐term care, at least not from the perspective of care professionals. In some ways, it might even work against mitigating the problem of dependency, since dealing with dependency sometimes means getting closer to the care recipient, rather than more distant. This became apparent in our discussion of equalizing goods, which seek to tighten the bond between caregiver and recipient. Paternalism, domination or subordination is only one type of problem tackled by care professionals in their dependency work; and tackling these problems may be balanced against tackling the other problems of dependency—diminished parity and diminished self‐worth.

The relationship between agentive, equalizing and affirmative goods is still more complex, however. While not always compatible, they could equally well be complementary in specific cases. This was evident in our discussion of affirmative goods, which aim for a sense of confidence, but may also indirectly tackle problems of diminished self‐determination. We can imagine other ways in which the pursuit of these goods might have secondary effects on other problems of dependency: for instance, that tackling problems of parity might lead to increased confidence amongst residents. It seems that ‘conflict’ is only one way in which the goods in care practices might relate; in the case of the goods we traced here, their relation is also one of occasional complementarity. This is the third conclusion we draw from our material.

Another caveat is in order here. We have focused on care practices and viewed dependency from the perspective of care professionals. This may seem odd, since the experience of the problem of dependency will usually be the care recipient's—in our case, the resident with ID. What surfaces as a problem for professionals may not be what surfaces as a problem for them. Our aim was not to give a phenomenological account of experiencing dependency as a problem, which is a project others have taken up (Eriksson & Andershed, 2008; Piredda et al., 2015; Strandberg et al., 2000; Strandberg, Norberg, & Jansson, 2003). Instead, we provide ideas about how dependency is treated as a problem by care professionals. Nonetheless, dependency work is a reciprocal process to a degree we have not been able to address here. In calibrating between goods (and how to best give shape to them), professionals engage in a two‐way process with the people for whom they care. This collaboration requires tinkering with conflicting wishes, needs and goods (Mol, Moser, & Pols, 2010). It can only be successful if people with ID can contribute to shaping the solution.

None of what we have argued is meant to suggest that dependency is exclusively or primarily a *problem* for long‐term care. Dependency, after all, is part of being human. We simply contend that when dependency *does* surface as a problem (as it often does in relations of long‐term care), the problem frame of self‐determination is too narrow, as it fails to illuminate problems of parity and of self‐worth—and that these different problems ask for different solutions.

## CONFLICT OF INTEREST

All authors declare that there is no conflict of interest.

## Footnotes

^1^ Our discussion of care here is limited to what Kari Waerness (1984) calls ‘necessary care’, which the recipient cannot provide for herself. We thus exclude what she calls ‘personal service’, in which the care recipient outsources care to another she could provide to herself. As Tronto (2013, p. 22) notes, in the latter case, power is distributed differently from the situations we sketch here, as the care recipient, who commands the care work, wields greater power than the caregiver.

^2^ For examples of this tendency, see Callus et al. (2019), Hamilton et al. (2017), and Williams et al. (2009).

^3^ The fieldwork was conducted in 2017 and 2018 in 12 group homes of seven different care providers. We shadowed 13 care professionals for one or two days, as well as 28 residents for one or two days. The study was submitted to the Medical Ethical Review Board of the Utrecht Medical Center for ethical approval. The board decided that no ethical approval for the study was required under the Dutch Medical Research Involving Subjects Act, meaning we could begin the study (Ref. WAG/mb/17/017624). Approval for the study was granted by all participating care providers prior to data collection. All participants were recruited according to principles of informed consent and signed a consent form (in the case of residents with severe ID, signing occurred by proxy).

^4^ All names are pseudonyms.
